# Pax6 loss alters the morphological and electrophysiological development of mouse prethalamic neurons

**DOI:** 10.1242/dev.200052

**Published:** 2022-03-17

**Authors:** Tian Tian, Idoia Quintana-Urzainqui, Zrinko Kozić, Thomas Pratt, David J. Price

**Affiliations:** 1 Simons Initiative for the Developing Brain, Hugh Robson Building, George Square, Edinburgh EH8 9XD, UK; 2 Developmental Biology Unit, European Molecular Biology Laboratory (EMBL), Meyerhofstrasse 1, 69012 Heidelberg, Germany

**Keywords:** Pax6, Transcription factors, Prethalamus, Neuronal morphogenesis, Axon initial segment, Neuronal activity

## Abstract

Pax6 is a well-known regulator of early neuroepithelial progenitor development. Its constitutive loss has a particularly strong effect on the developing prethalamus, causing it to become extremely hypoplastic. To overcome this difficulty in studying the long-term consequences of Pax6 loss for prethalamic development, we used conditional mutagenesis to delete Pax6 at the onset of neurogenesis and studied the developmental potential of the mutant prethalamic neurons *in vitro*. We found that Pax6 loss affected their rates of neurite elongation, the location and length of their axon initial segments, and their electrophysiological properties. Our results broaden our understanding of the long-term consequences of Pax6 deletion in the developing mouse forebrain, suggesting that it can have cell-autonomous effects on the structural and functional development of some neurons.

## Abstract (precis)

**Summary:** Pax6 impacts neurite extension, axon initial segment properties and the ability to fire normal action potentials in maturing neurons, revealing actions extending beyond those previously characterised in progenitors.

## INTRODUCTION

The early neuroepithelium is patterned by the regional expression of transcription factors that specify the subsequent development of each region. The extent to which the actions of these transcription factors influence the later development of the functional properties of neurons is much less clear.

We studied the transcription factor Pax6, a well-known regulator of early neural development (Cvekl and Callaerts, 2017). Pax6 expression in the mouse neuroepithelium is first detected soon after neural tube closure and continues in specific forebrain progenitors, where it has pivotal functions in diverse early developmental processes (Hanson and Van Heyningen, 1995; Engelkamp et al., 1999; Mi et al., 2013; Manuel et al., 2015; Cvekl and Callaerts, 2017). In most of these regions, expression of Pax6 is lost in cells exiting the cell cycle during neurogenesis (Duan et al., 2013). Most, if not all, prethalamic progenitors are Pax6-positive but, unusually, strong Pax6 expression is retained by many post-mitotic neurons in the embryonic prethalamus (Duan et al., 2013; Caballero et al., 2014). Constitutive deletion of Pax6 all but prevents the formation of the prethalamus, precluding an analysis of the consequences of its loss for developing prethalamic neurons (Stoykova et al., 1996; Warren and Price, 1997).

We re-examined our previously-reported RNAseq dataset of changes in prethalamic gene expression following acute Pax6 deletion at the onset of neurogenesis (Quintana-Urzainqui et al., 2018) and found upregulated expression of genes involved in neuronal morphogenesis and ion transport. We investigated the effects of acute Pax6 deletion on the ability of prethalamic neurons to acquire normal structural and functional properties. We used dissociated culture, thereby increasing the likelihood of detecting cell-autonomous effects. We found that the neurites of Pax6-deleted prethalamic neurons grew at abnormal speed, their axon initial segments (AISs) tended to be longer and to extend further from the soma, and their electrophysiological properties were altered. Our results indicate that, in addition to its role in early prethalamic progenitors, Pax6 is also required in the later structural and functional development of prethalamic neurons.

## RESULTS AND DISCUSSION

### Prethalamic Pax6 deletion caused upregulation of genes involved in neuronal morphogenesis and ion transport

**Fig. 1.**
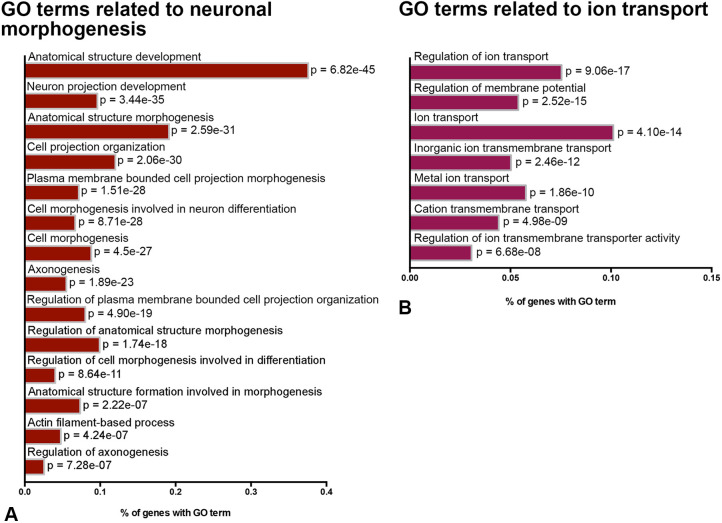
**Pax6 deletion in the prethalamus caused upregulated expression of genes involved in neuronal morphogenesis and ion transport-related GO terms.** (A) The top 14 most highly enriched, non-redundant GO terms related to neuronal morphogenesis and (B) the top seven most highly enriched, non-redundant GO terms related to ion transport in the Pax6 cKO prethalamus. See also Table S1.

At E13.5, Pax6 is expressed in cortical, thalamic and prethalamic progenitors, and in a population of prethalamic neurons (Fig. S1A,A′). We interrogated an existing RNAseq dataset showing significant transcriptional changes (adjusted *P*<0.05) in E13.5 prethalamus after acute deletion of Pax6 from E11.5, which is after prethalamic neuroepithelial specification and around the time prethalamic neurogenesis starts (Quintana-Urzainqui et al., 2018). Gene ontology (GO) term enrichment analysis on these genes revealed 495 upregulated and 125 downregulated GO terms (Table S1). Among the top 200 upregulated GO terms, 25 related to neuronal morphogenesis and 12 to ion transport. After removing child terms, 14 GO terms were related to neuronal morphogenesis and seven were related to ion transport (Fig. 1A,B, Table S1).

Pax6 loss upregulated genes important for the actin and microtubule cytoskeleton during neuritogenesis, for axon specification and for neurite elongation (Table S2). In other systems, upregulation of these effector genes increases actin nucleation, actin bundling, microtubule assembly and bidirectional intracellular transportation, which have been shown to augment axon/neurite elongation (Witte and Bradke, 2008; Stiess and Bradke, 2011; Flynn, 2013; Sainath and Gallo, 2015). Pax6 loss also upregulated genes involved in relaying extracellular signals to the actin and microtubule cytoskeleton, and genes encoding several components of the PAR3/6 complex, which plays a vital role during the establishment of neuronal polarity (Table S2) (Nishimura et al., 2004; Shi et al., 2004; Barnes and Polleux, 2009; Lalli, 2014).

The AIS is located at the most proximal end of the axon and is where action potentials usually initiate (Kole and Stuart, 2008; Kole et al., 2008; Rasband, 2010; Leterrier, 2018). Genes encoding the cytoskeletal components of the AIS were also upregulated (Table S2), including the cytoskeletal scaffold protein AnkyrinG (AnkG, also known as Ank3) and βIV Spectrin (Sptbn4). In neurons, AnkG is restricted to the AIS and nodes of Ranvier, where it tethers high densities of specific types of voltage-gated ion channels and anchors itself to the underlying actin cytoskeleton via βIV Spectrin (Rasband, 2010; Leterrier, 2018). Genes encoding voltage-gated sodium channels (VGSCs) that show concentrated distributions within the AIS were also upregulated, as were genes that encode various voltage-gated ion channels expressed within the somatodendritic domain (Lai and Jan, 2006; Hu et al., 2009).

Based on this analysis, we hypothesised that conditional Pax6 deletion affects the morphogenesis, AIS formation and activity of prethalamic neurons. To test these hypotheses, we measured the effects of the same conditional mutation as in the RNAseq study in an *in vitro* culture system.

### Pax6 loss caused defects in neurite extension in developing prethalamic neurons

Our protocol for tamoxifen-induced deletion caused Pax6 protein loss from E11.5 onwards in conditional knockouts (Pax6cKOs, *CAG^CreER^ Pax6^fl/fl^*) (Quintana-Urzainqui et al., 2018). No Pax6 protein was detected at the time of dissociation at E13.5 (Fig. S1B,B′). Littermates that were heterozygous for the *Pax6^fl^* allele were used as controls (Ctrl, *CAG^CreER^ Pax6^fl/+^*), as they continue to express Pax6 normally (Simpson et al., 2009).

**Fig. 2.**
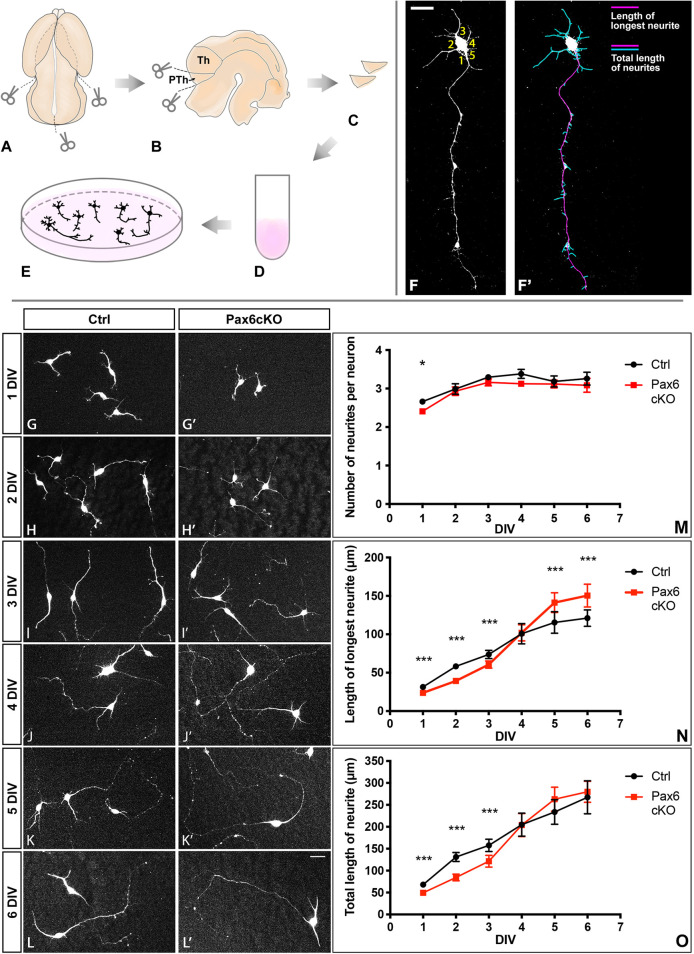
**Neuronal morphogenesis of prethalamic neurons *in vitro*.** (A-E) Schematic summary of E13.5 prethalamus dissection for dissociated cell culture. (F,F′) Example of how the number of neurites (F), the length of the longest neurite and the total length of neurites (F′) were measured for each neuron. Scale bar: 20 μm. (G-L′) Example of prethalamic neuron morphology labelled with Tuj1 from both genotypes on each DIV. Scale bar: 10 μm. (M-O) Quantification of the number of neurites (M), length of longest neurite (N) and total length of neurites (O) in Ctrl and Pax6cKO prethalamic neurons cultured for 1-6 DIV. Mixed-effect model; at least 100 neurons from each genotype were collected from each of three litters on each of the six DIVs. ***P<0.001, **0.001<*P*<0.01, *0.01<*P*<0.05. Data are mean±s.e.m.

Fig. 2A-E shows the process of E13.5 prethalamic dissection for dissociated cell culture. On each day *in vitro* (DIV), we measured: the number of neurites; the length of the longest neurite; and the total length of neurites (Fig. 2F,F′). Most cultured Ctrl and Pax6cKO prethalamic cells were positive for Tuj1 and neuritogenesis had begun at 1 DIV (Fig. 2G,G′). Most prethalamic neurons displayed one longest neurite, presumably the developing axon, and several shorter neurites (Fig. 2G-L′). Pax6cKO prethalamic neurons had fewer neurites than Ctrl neurons at 1DIV (*P*=7.20×10^−3^, *n*=3, the statistical test is always a mixed-effect model, and the unit of *n* is always cultures from separate litters unless stated otherwise) but not after longer culture (Fig. 2M). The longest neurites and the total neurite lengths in Pax6cKO prethalamic neurons were shorter than in Ctrls from 1-3 DIV (Fig. 2N,O). However, after 3 DIV, the longest neurites elongated more rapidly in Pax6cKO than in Ctrl neurons, and they became significantly longer at 5DIV (*P*=2.17×10^−5^, *n*=3) and 6DIV (*P*=1.66×10^−6^, *n*=3; Fig. 2N). Thus, Pax6cKO prethalamic neurons developed a relatively normal complement of neurites after a delayed start, the longest of which later outstripped their equivalents in Ctrl neurons. Mechanisms limiting axon elongation might be important because most prethalamic neurons project only short distances (Martinez-Ferre and Martinez, 2012; Willis et al., 2015), unlike neighbouring thalamic neurons, many of which project long thalamocortical axons.

It is unclear what causes delayed neuronal morphogenesis in Pax6cKO prethalamic neurons early in cell culture. Previous studies have proposed that the establishment of neuronal polarity can be a speed-limiting step of neuronal morphogenesis *in vitro* (Bradke and Dotti, 2000; Barnes and Polleux, 2009; Yogev and Shen, 2017). We examined this further, as the RNAseq analysis indicated that Pax6cKO prethalamic cells upregulated expression of genes encoding components of the Par3/6 complex. We used the localised distribution of Par3 as an indicator of neuronal polarity (Nishimura et al., 2004). Our results showed no significant differences in the amount and distribution of Par3 within the neurites and the soma between Ctrl and Pax6cKO prethalamic neurons at 3 DIV (Fig. S2A-N). Further analysis on neurite organisation and branching showed no significant differences between Ctrl and Pax6cKO neurons (Fig. S2O,P). We concluded that the establishment of neuronal polarity was not affected in *Pax6*-deleted prethalamic neurons.

### Pax6 loss altered AIS length and location in developing prethalamic neurons

**Fig. 3.**
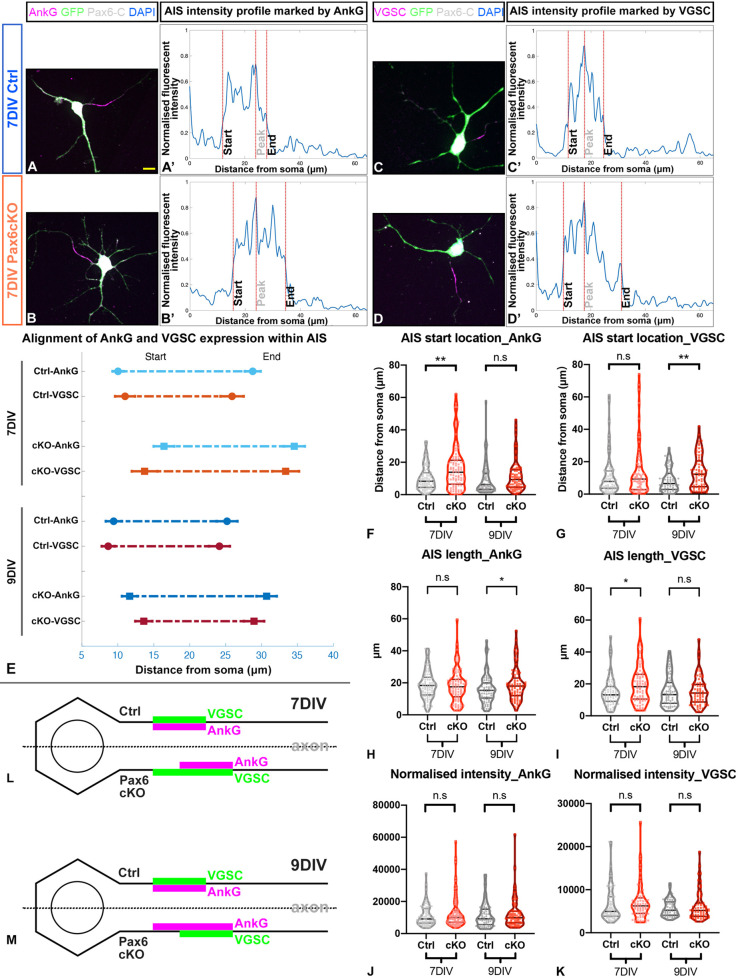
**Loss of Pax6 changed the length and location of the AIS in the prethalamic neurons.** (A-D) Examples of immunohistochemistry staining showing localised distribution of AnkG or VGSC, marking the AIS, in prethalamic neurons cultured for 7 DIV. Scale bar: 10 μm. (A′-D′) Examples of normalised fluorescence intensity of AnkG or VGSC along the axon, termed AIS intensity profile. The peak is where the normalised fluorescence intensity is highest. The start location (close to the soma) and the end location are where normalised fluorescence intensities fall to 33% of peak (Grubb and Burrone, 2010). (E) Alignment of the AIS length and location marked by the two markers (AnkG and VGSC) in Ctrl and Pax6cKO prethalamic neurons cultured for 7 and 9 DIV. Comparison of the start location (F,G), length (H,I) and normalised intensity (J,K) of the AIS marked by either AnkG or VGSCs in Ctrl and Pax6cKO prethalamic neurons cultured for 7 and 9 DIV. AIS length is the distance between the start and the end location. (L,M) Schematic summary of changes of AIS length and location in Pax6cKO prethalamic neurons at 7 and 9 DIV. Mixed-effect model, at least 30 neurons from each genotype were collected from each litter on either 7 or 9 DIV. Three litters in total (*n*=3). ***P<0.001, **0.001<*P*<0.01, *0.01<*P*<0.05. (E) Data are mean±s.e.m. (F-K) Data are median with interquartile range.

We investigated AIS formation with immunohistochemistry for AnkG and VGSC in dissociated prethalamic neurons at 7 and 9 DIV. In these experiments, we used an antibody that recognises an epitope in the C-terminal domain of Pax6 (referred to in Fig. 3A-D as Pax6-C), which is produced even in Pax6cKO neurons due to translation from preserved internal initiation sites (see Materials and Methods; Kammandel et al., 1999; Simpson et al., 2009). This confirmed that we were comparing Ctrl and Pax6cKO neurons that expressed the *Pax6* gene (although the latter did not generate full-length functional Pax6 protein: Fig. S1B,B′).

At both 7 DIV and 9 DIV, most prethalamic neurons of both genotypes developed a single AIS (Fig. 3A-D, magenta segments) where the profiles of AnkG and VGSC expression (quantified as illustrated in Fig. 3A′-D′) coincided at the proximal end of a neurite (supporting our conclusion that the establishment of neuronal polarity was unaffected in Pax6cKO prethalamic neurons). AISs tended to extend further from the soma in Pax6cKO neurons (7 DIV AnkG, *P*=2.94×10^−3^; *n*=3; 9 DIV VGSC, *P*=4.56×10^−3^, *n*=3; Fig. 3E-G). Average AIS lengths measured with VGSC at 7 DIV (*P*=0.0140, *n*=3) and with AnkG at 9 DIV were significantly higher in Pax6cKO neurons (*P*=0.0263, *n*=3, Fig. 3H,I). In no case were densities of staining (i.e. the average fluorescence intensity per unit length of neurite) with either marker different between the two genotypes (Fig. 3J,K). Fig. 3L-M summarises the lengthening and distal extension of the AIS in prethalamic neurons after 7 and 9 DIV.

AIS assembly is considered an intrinsic property of neurons, requiring no extracellular or glial-dependent cues (Ogawa and Rasband, 2008). The observed AIS lengthening was in line with the upregulated expression of VGSCs and AnkG in the RNAseq data, but their distal shift was unexpected. As the master regulator of AIS assembly, AnkG specifies AIS formation and VGSC clustering (Zhou et al., 1998; Rasband, 2010), but little is known about the mechanisms that contribute to the enrichment and targeting of AnkG to the proximal axon and specify AIS location (Rasband, 2010; Berger et al., 2018; Leterrier, 2018).

### Loss of Pax6 affected the electrophysiological properties of prethalamic neurons

As the reported changes of the AIS and the expression of voltage-gated ion channels might change neuronal excitability and electrical functions (Grubb and Burrone, 2010; Grubb et al., 2011; Kaphzan et al., 2011; Höfflin et al., 2017; Booker et al., 2020), we performed whole-cell patch-clamping on prethalamic neurons cultured for 7 and 9 DIV.

**Fig. 4.**
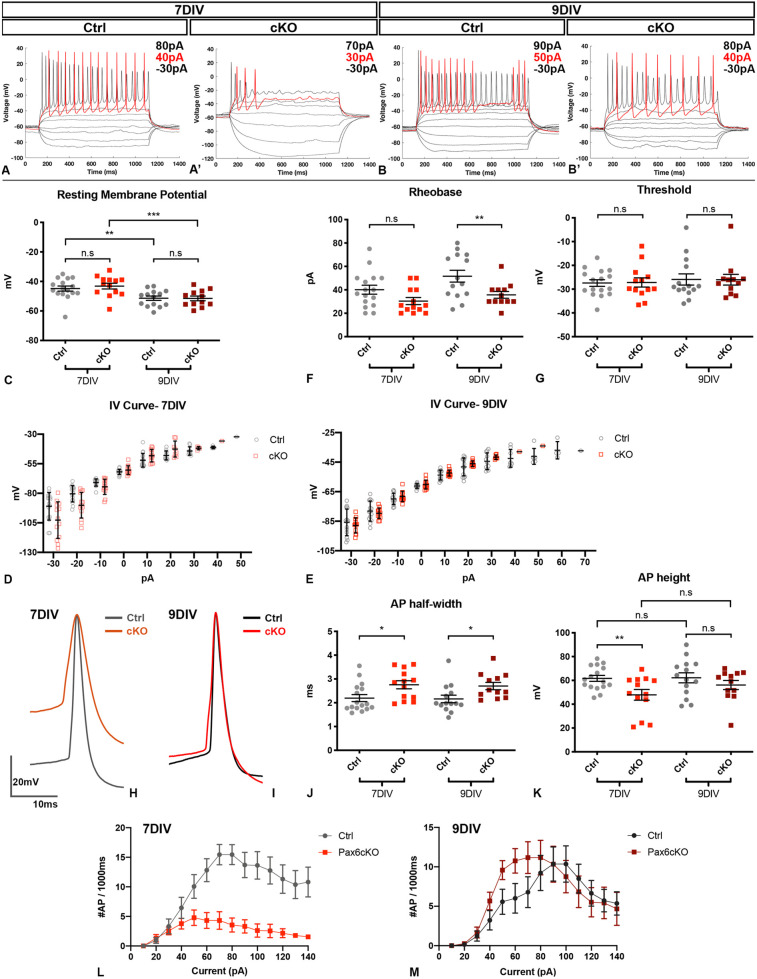
**Pax6cKO prethalamic neurons fire APs differently at 7 and 9 DIV.** (A-B′) Example of membrane potential changes of Ctrl and Pax6cKO prethalamic neurons responding to depolarising current steps (−30pA to 140 pA, in 10pA increments, 1000 ms). Red traces: membrane potential traces at rheobases. (C,F,G) Comparison of resting membrane potential, rheobase and threshold potential in Ctrl and Pax6cKO prethalamic neurons cultured for 7 and 9 DIV. (D,E) IV curves in Ctrl and Pax6cKO prethalamic neurons cultured for 7 and 9 DIV. (H,I) Aligned and overlayed traces of the first APs fired at rheobases by the Ctrl and Pax6cKO prethalamic neurons cultured for 7 and 9 DIV. (J,K) Comparison of the half-width and height of the first AP at rheobases in Ctrl and Pax6cKO prethalamic neurons cultured for 7 and 9 DIV. (L,M) Current frequency plot for Ctrl and Pax6cKO prethalamic neurons cultured for 7 and 9 DIV, indicating differences in AP firing frequency in response to applied current steps. Mixed effect model, 16 neurons from Ctrl at 7 DIV, 13 neurons from Pax6cKO at 7 DIV, 14 neurons from Ctrl at 9 DIV, 12 neurons from Pax6cKO at 9 DIV. four litters for each genotype at both DIVs (*n*=4). ***P<0.001, **0.001<*P*<0.01, *0.01<*P*<0.05. (C-G,J-K) Data are mean±s.e.m. (D,E) Data are mean±s.d.

To induce action potentials (APs), we stimulated the prethalamic neurons with small depolarising current steps from −60 mV. Prethalamic neurons of both genotypes at both ages were able to fire APs (Fig. 4A-B′). From 7 to 9 DIV, resting membrane potentials (RMPs) became significantly more negative in both the Ctrl (*P*=4.94×10^−3^, *n*=4) and Pax6cKO prethalamic neurons (*P*=9.55×10^−4^, *n*=4), as expected in maturing neurons (Linaro et al., 2019), with no significant differences between genotypes (7 DIV, *P*=0.515, *n*=4; 9 DIV, *P*=0.944, *n*=4, Fig. 4C). Fig. 4D,E showed how membrane potentials changed in response to specific negative (hyperpolarising) and positive (depolarising) current inputs before rheobases were reached. At 7 DIV but not at 9 DIV, negative current inputs hyperpolarised membrane potentials significantly more in Pax6cKO than in Ctrl neurons (*P*=2.00×10^−4^, *n*=4, Fig. 4D,E). Although no differences were found at 7 DIV (*P*=0.0549, *n*=4), rheobase became significantly lower in Pax6 cKOs at 9 DIV (*P*=7.70×10^−3^, *n*=4, Fig. 4F). The AP threshold remained unchanged at both ages (7 DIV, *P*=0.893, *n*=4; 9 DIV, *P*=0.945, *n*=4, Fig. 4G).

We examined the waveforms of the first APs fired by the Ctrl and the Pax6cKO prethalamic neurons in response to the current stimulus at their rheobases. Fig. 4H,I shows the overlaid example traces of the first APs shown in Fig. 4A-B′. AP halfwidth was significantly broader in Pax6cKOs at both ages (7 DIV, *P*=0.0125, *n*=4; 9 DIV, *P*=0.0102, *n*=4) and AP height was significantly lower in Pax6cKOs at 7 DIV (*P*=4.78×10^−3^, *n*=4, Fig. 4J,K).

Prethalamic neurons from both genotypes were able to fire multiple APs after rheobases were reached but, at 7 DIV, Pax6cKO prethalamic neurons showed a significant reduction in maximum AP number (Fig. 4L, *P*=1.12×10^−5^, *n*=4). The AP firing frequency in the Pax6cKO neurons at 7 DIV was 7±1.4 Hz in comparison with 18.6±1.9 Hz in the Ctrl neurons. This phenotype was recovered at 9 DIV, when Pax6cKO and Ctrl neurons fired comparable maximum numbers of APs (Fig. 4M, *P*=0.897, *n*=4). At 9 DIV, Pax6 loss also caused a leftward shift in the current-frequency response (Fig. 4M), indicating that a similar number of APs were generated using a lower amplitude of current stimulus in the Pax6cKO neurons. Therefore, Pax6 loss can affect the activity of the prethalamic neurons, resulting in changes in their excitability level, the waveforms of somatic APs and their ability to fire APs repetitively.

The distal shift of the AIS in Pax6cKO neurons might have contributed to the widening of the AP waveforms recorded from their soma as these recordings would have been mainly the backpropagated APs generated at the AIS (Kole et al., 2007), and increasing the distance between the AIS and the soma would increase voltage attenuation, resulting in wider and slower APs. The underlying causes for the decrease in rheobase in the Pax6cKO prethalamic neurons are less clear. Changes in AIS length and location could be a factor as previous studies have shown that both increased length and distal relocation of the AIS can promote excitability (Grubb and Burrone, 2010; Buffington and Rasband, 2011; Kaphzan et al., 2011; Höfflin et al., 2017; Jamann et al., 2018; Goethals and Brette, 2020). However, changes of AIS geometry alone are unlikely to provide a full explanation, as theoretical modelling suggests that they would produce a modest decrease (a few mV) in threshold potential (Goethals and Brette, 2020), for which we found no evidence. Therefore, it is likely that changes outside the AIS contributed to the decrease in rheobase in Pax6cKO prethalamic neurons. The surprising observation that 7 DIV Pax6cKO prethalamic neurons fired significantly fewer APs and entered the state of depolarisation block with significantly lower amplitude of current stimulus, despite having significantly longer AISs, might be explained in two ways. First, there might be AIS defects; second, RNAseq data suggested upregulation of several voltage-gated potassium channels belonging to the Kv4 family [Kv4.1(KCND1), Kv4.2(KCND2) and Kv4.3(KCND3), Table S2], which can efficiently reduce repetitive firing (Fransén and Tigerholm, 2010; Hermanstyne et al., 2017; Kim et al., 2020).

In conclusion, our results suggest that Pax6 loss from the prethalamic neuroepithelium causes the generation of neurons with an abnormal developmental potential. Mutant prethalamic neurons show subtly disrupted axonal extension, abnormal AISs and somatic AP waveforms and excitability, which might impact on prethalamus-thalamus circuit formation and might contribute to defective nervous system development in PAX6-deficient humans (Kikkawa et al., 2019).

## MATERIALS AND METHODS

### Experimental model and subject details

#### Mice colony maintenance and transgenic lines

Mouse lines used to generate tamoxifen-induced deletion of Pax6 throughout the embryo were as described previously (Quintana-Urzainqui et al., 2018). Pregnant mice were given 10 mg of tamoxifen (Sigma-Aldrich) by oral gavage on embryonic day 9.5 (E9.5) to induce Pax6loxP deletion, and embryos were collected on E13.5. Embryos heterozygous for the Pax6flox allele (Pax6^fl/+^; CAGG^CreER^) were used as controls (Ctrl), as previous studies have shown no detectable defects in the forebrain of Pax6^fl/+^ embryos (Simpson et al., 2009). Embryos carrying two copies of the floxed Pax6 allele (Pax6^fl/fl^; CAGG^CreER^) were the experimental conditional knockout (cKO) group.

For staging of embryos, the first day the vaginal plug was detected was considered as embryonic day 0.5 (E0.5).

All animal husbandry was conducted in accordance with the UK Animal (Scientific Procedures) Act 1986 regulations and all procedures were approved by Edinburgh University's Animal Ethics Committee.

### Method details

#### Dissociated prethalamic cell culture preparation

##### Dissection of the prethalamus

Dissection of the prethalamus at E13.5 was first practised extensively using the DTy54 transgene mouse line, as specified previously (Quintana-Urzainqui et al., 2018). Guidance by the GFP expression, which marked Pax6-expressing cells, allowed us to identify reliable morphological landmarks. To dissect the prethalamus, Pax6^fl/+^; CAGG^CreER^ and Pax6^fl/fl^; CAGG^CreER^ embryos from E13.5 were collected and decapitated. The neural tube was separated from the epidermal and mesodermal tissue and cut in half along the dorsal and ventral midline (Fig. 2A,B). From E9.5, morphological segmentation of the diencephalon starts and the diencephalic prosomeres become apparent from E10-E11, as ventricular ridges and lateral wall bulges appear (Vieira et al., 2009). These morphological landmarks were used to distinguish prethalamus from the surrounding tissue (the thalamus and the eminentia thalami) during dissection (Fig. 2C).

##### Dissociated cell culture

After being cut out, the prethalamus from the two halves of the neural tube of the same embryo were put together and chopped into smaller pieces for dissociated cell culture using the Papain Dissociation System (Worthington Biochemical) according to the manufacturer's protocol (Fig. 2C-E). The prethalamus from each embryo was dissociated and cultured individually. The number of cells obtained after dissociation was measured using a haemocytometer. Additionally, Trypan Blue staining was used to determine the ratio of viable to damaged cells. To adjust the plating density of the cell culture, cells obtained after dissociation were resuspended using a predetermined amount of culture medium [Advanced DMEM/Neurobasal medium 1:1, supplemented with N2 (100×) and B27 (50×) neural supplement, Thermo Fisher Scientific]. 130μl of the culture medium containing the desired number of resuspended cells was then added onto the 9 mm circular coverslips (Thermo Fischer Scientific) coated with Poly-L-lysine and laminin (Thermo Fisher Scientific). Owing to the surface tension of the culture medium, the culture medium containing the dissociated cells would stay within and fill up the realm of the coverslips. The cell cultures were then incubated at 37°C with 5% CO_2_ for 1 h to allow the cells to attach to the coverslips. In this way, all the cells obtained after the dissociation were retained within the coverslip, and the exact cell density of plating could be calculated using the total amount of cells divided by the surface areas of the coverslips being used. The plating density of prethalamic cell culture used for studying neuronal morphogenesis and AIS formation was 20 cells/mm^2^, and the plating density of prethalamic cell culture used for electrophysiological recordings was 600 cells/mm^2^. After 1 h, 240 μl of the culture medium was then added into each well of the 48-well plate (Greiner Bio-One) that contains the coverslip. The dissociated prethalamic cells were cultured for 1-9 days. Light microscopy was used to monitor the condition of cell cultures daily.

#### Genotyping

We dissected tissue from the tails of each embryo, extracted DNA and performed PCR amplification to detect the alleles of interest. For the detection of the floxed Pax6 allele, PCR reaction was performed in a final volume of 25 μl containing 1.5 μl of extracted DNA, 0.5 mM primer mix (Simpson et al., 2009; forward primer, 5′-AAA TGG GGG TGA AGT GTG AG-3′; reverse primer, 5′-TGC ATG TTG CCT GAA AGA AG-3′), 0.5 mM dNTPs mix, 1× PCR reaction buffer and 5 U/μl Taq DNA Polymerase (Qiagen). PCR was performed with 35 cycles and a Tm of 59°C. The PCR product was subsequently run in a 2% agarose gel. Wild-type allele results in a fragment of 156 bp and the floxed allele fragment was 195 bp; therefore, two bands indicated the heterozygous condition (used as controls) and one strong 195 bp band identified the homozygous floxed allele condition (Pax6cKOs).

#### Histological processing and imaging

##### Sample processing for immunohistochemistry

Cryosections were obtained following the methods described by Quintana-Urzainqui et al. (2018). Cell culture samples were obtained by removing the coverslips containing the prethalamic neurons from the culture medium. 1× phosphate-buffered saline (PBS) warmed to 37°C were used to rinse the prethalamic neurons three times. For cell culture samples used in the neuronal morphology and polarity studies, the prethalamic neurons were fixed in 4% PFA for 20 min. For cell culture samples used in the AIS study, the prethalamic neurons were fixed in 2% PFA/4% sucrose for 10 min to prevent degradation of AnkG protein by the fixative. The cell culture samples were further rinsed with 1×PBS and kept in 1× PBS at 4°C until processed.

##### Fluorescent immunohistochemistry

Fluorescent immunohistochemistry on cryosections were performed as described previously (Quintana-Urzainqui et al., 2018). Fluorescent immunohistochemistry on cell culture were performed as follows: fixed prethalamic neurons were rinsed with 0.1% Triton X-100 in 1×PBS (0.1% PBST) and permeabilised in 0.5% Triton X-100 in 1×PBS (0.5% PBST) for 10 min. Then cells were washed with 0.1% PBST three times and further blocked with blocking solution (20% goat or donkey serum in 0.1% PBST) for 2 h. The blocking serum containing primary antibodies was added to the cells for overnight incubation. On the second day, cells were washed with 0.1% PBST and further incubated with blocking serum containing the corresponding secondary antibodies (Streptavidin Alexa Fluor 488, 546 or 647 conjugates; Thermo Fisher Scientific) for 1 h. Cells were then washed with 1×PBS and further incubated with DAPI (Thermo Fisher Scientific) for counterstaining of the nucleus. The coverslips were mounted in ProLong Gold Antifade Mountant (Thermo Fisher Scientific) for further imaging.

Mouse monoclonal anti-Pax6 (1:10) was used to detect the N-terminal domain of Pax6 that is absent in the Pax6^fl/fl^; CAGG^CreER^ embryos (Simpson et al., 2009). Rabbit polyclonal anti-Pax6 (1:200) was used to identify dissociated Ctrl and Pax6cKO prethalamic neurons that expressed the *Pax6* gene as this antibody recognises an epitope in the C-terminal domain of Pax6, which is produced even in Pax6cKO neurons due to translation from preserved internal initiation sites ((Kammandel et al., 1999; Simpson et al., 2009). Antibody details can be found in Table S3.

##### Imaging

Fluorescence images of the dissociated cells in the neuronal morphogenesis study were taken using a Leica DM5500B automated upright microscope connected to a DFC360FX camera. The route of acquiring images started from the upper-left corner towards the lower-right of the 9 mm circular coverslip. Every image being taken was from an adjacent visual field of the previous image to cover as many cells on the coverslip as possible and without imaging the same cells twice. Fluorescence images of the dissociated cells in the PAR3 study were taken using the Zeiss LSM800 confocal microscope with Airy Scan. Fluorescence images of the dissociated cells in the AIS study were taken using the Andor Revolution XDi Spinning disk confocal microscope.

#### Whole-cell patch-clamp recording

For electrophysiological recordings on the dissociated prethalamic neurons cultured for 7 and 9 DIV, coverslips with attached prethalamic neurons were transferred to a submerged recording chamber perfused with carbonated ACSF [in mM: 150 NaCl, 2.8 KCl, 10 HEPES, 2 CaCl2, 1 MgCl2, 10 Glucose (pH7.3)], at a flow rate of 4-6 ml/min at 22-23°C. Prethalamic neurons were visualised with a digital camera (SciCam Pro, Scientifica) mounted on an upright microscope (BX61-WI, Olympus) and a 40× water-immersion objective lens (1.0 N.A., Olympus). Whole-cell patch-clamp recordings were performed with a Multiclamp 700B amplifier (Molecular Devices), filtered at 10 kHz with the built-in 4-pole Bessel Filter and digitised using a Digidata 1440A digitiser board (Molecular Devices) at 20 kHz. Recording pipettes were pulled from borosilicate glass capillaries (Harvard Apparatus, 30-0060) on a horizontal electrode puller (P-97, Sutter Instruments) to a tip resistance of 4-6 MΩ. Recording pipettes were filled with K-gluconate-based internal solution [in mM: 130 K-gluconate, 4 Glucose, 10 HEPES, 0.1 EGTA, 0.025 CaCl2, 20 sucrose (pH=7.2), 290-300mOsm]. Recording pipettes were positioned with a micromanipulator (Scientifica PatchStar). Data were acquired from cells with access resistance <25MΩ. pClamp 10 (Axon Instruments) was used to generate the various analogue waveforms to control the amplifier and record the traces.

Resting membrane potential of the prethalamic neurons was recorded with current clamped with no current input (I=0 pA) for 30 s. Constant current was injected to hold the cells close to −60 mV and I-V curve, rheobase and threshold was assessed by current injections from −30 to +140 pA for 1000 ms (10 pA steps). All AP properties were determined from the first AP elicited at rheobase. Analysis of electrophysiological data was performed offline using a custom-written Matlab script kindly provided by Dr Adam Jackson (Weill Institute for Neurosciences, University of California, San Francisco, CA, USA), blind to genotype.

#### Image analysis

##### Measurement of neurite length

Measurement of neurite length was performed using the freehand line tool in the FIJI package of ImageJ. Whole morphology of the neurons was marked by fluorescent immunohistochemistry reacting with the Tuj1 antibody, which labels the neuron-specific Class III β-tubulin of the cytoskeleton. For each Tuj1-positive cell, three parameters – the number of neurites, the length of the longest neurite and the total length of neurites – were measured. A neurite was defined as a stable protrusion from the soma with strong Tuj1 staining. Protrusions from the soma that were thin and had faint Tuj1 staining were considered as either artefacts or filopodia, which mainly consist of F-actin and were not considered as neurites. The length of a neurite was measured by tracing down the neurite from the edge of the soma to the most distal edge of Tuj1 staining. Such measurement was performed for every neurite of each neuron analysed. The length of the longest neurite was the highest reading among these measurements of each neuron. All the protrusions that were positive for Tuj1 staining and stemmed from the neurites were considered as branches. The length of each branch was measured from where it stemmed from the neurite to its furthest edge of Tuj1 staining. The total length of neurites for each neuron was calculated as the sum of the length of all the neurites and branches.

##### Measurement of PAR3 distribution

Measurement was performed with the IMARIS software (Bitplane, version 9.1.2). Dissociated prethalamic neurons cultured for 3 DIV were marked by fluorescent immunohistochemistry reacting with antibodies for PAR3, GFP for whole-cell morphology and DAPI for counterstaining of the nucleus.

The surface function of IMARIS was used to create a new GFP channel. To do so, the signal intensity threshold was adjusted manually based on the specific situation of each neuron so that IMARIS was able to detect the space where the GFP signal is above that set threshold. This threshold was determined by fitting and adjusting different threshold values so that the GFP-positive space IMARIS detected optimally matched the actual cytoplasmic volume and included almost all the neurites and cytoplasmic protrusions. Places where their GFP signals were above this threshold but resided outside the cytoplasm were manually deleted. In this way, a new GFP channel was created, which only included and highly resembled the entire cytoplasm of the neurons. A new PAR3 channel was then created using the new GFP channel as the template of the cytoplasmic volume to exclude any Par3 staining outside of the cytoplasm. With this new PAR3 channel, we could then detect the highest intensity of PAR3 expression within the cytoplasm. Different thresholds were set for IMARIS to detect the cytoplasmic volumes in which the PAR3 intensities were above 75%, 50%, 25% and 10% of its own highest intensity value. This allowed us to quantify the cytoplasmic volume that had higher expression levels of PAR3 at each of these thresholds, and also to visualise the distribution of different intensities of PAR3 within the cytoplasm.

##### Sholl analysis and neurite branching analysis

Sholl analysis was performed as previously described (Thongkorn et al., 2021), but using the Sholl Analysis Plugin (https://github.com/morphonets/SNT) of ImageJ2 (Version 2.3.0/1.53f). Whole-cell morphology of dissociated prethalamic neurons cultured for 3 DIV was marked by fluorescent immunohistochemistry, reacting with antibodies to GFP for whole-cell morphology and with DAPI for counterstaining of the nucleus. The images of these neurons were taken with the Zeiss LSM800 confocal microscope with Airy Scan then converted into 2D images with maximum intensity projection on *z*-stack. A threshold was set to include as much as the whole morphology of each neuron. To construct the circles to perform the Sholl analysis, the centre of the soma was marked manually and circles of radii from 0 to 240 μm from the soma was constructed with 10 μm increment.

To count the number of branches per neuron, the surface function of IMARIS was used to create a new GFP channel for the whole-cell morphology of each neuron. The number of branches per neuron was counted as branch points plus the number of end branches that were GFP positive.

##### Measurement of the intensity profile of AIS

The intensity profile of AIS was generated by tracing down the neurite bearing the localised expression of either AnkG or VGSC, 80 μm from the edge of the soma, using the freehand line tool in the FIJI package of ImageJ, resulting in a graph with the level of AnkG or VGSC expression at each pixel from the soma corresponding to the specific distance from the soma.

### Quantification and statistical analysis

All experiments were performed blind to genotype. All data was analysed with either a linear mixed-effect model (LMM) or its generalised form (GLMM), unless stated otherwise. The variability due to random effects (animal, litter) was taken into account, allowing for the calculation of the genotype effect size. Where reported, statistical significance was assumed if *P*<0.05.

## Supplementary Material

Supplementary information

Reviewer comments
