# Supplementary material for: Pax6 loss alters the morphological and electrophysiological development of mouse prethalamic neurons
Source: Development. 2022 Mar 17;149(6):dev200052. doi: 10.1242/dev.200052 (PMC8977098; doi:10.1242/dev.200052)
Supplement: Supplementary information [file develop-149-200052-s1.pdf]

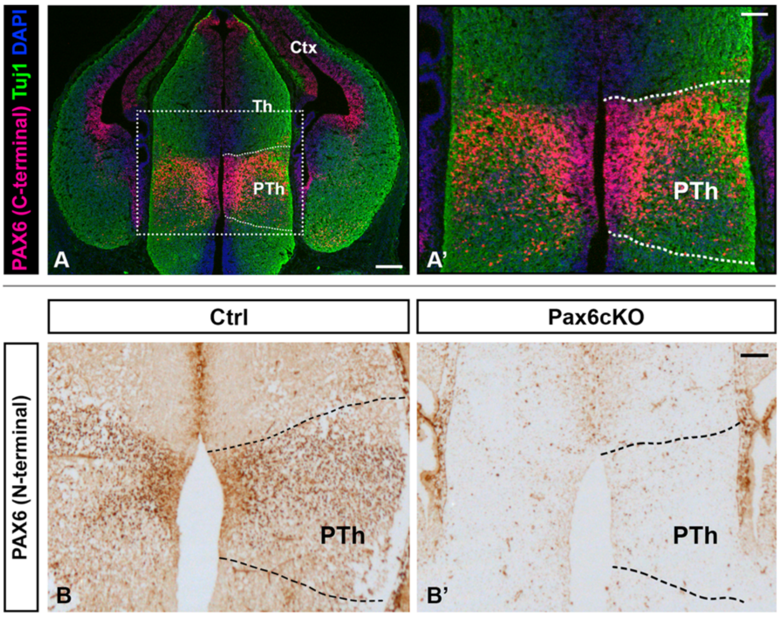

Fig. S1. (A-A') Immunohistochemistry showing Pax6 expression overlapping with Tuj1, a marker for postmitotic neurons, in the E13.5 prethalamus of the control littermate. PTh, prethalamus, Th, thalamus, Ctx, cortex. (B-B') Pax6 immunohistochemistry at E13.5 showing CAG^{CreER}-induced loss of Pax6 in the prethalamus following tamoxifen administration at E9.5. (C) The top 14 most highly enriched, non-redundant GO terms related to neuronal morphogenesis and (D) the top 7 most highly enriched, non-redundant GO terms related to ion transport in the Pax6 cKO prethalamus. Scale bar: (A) 250 μm; (A', B-B') 100μm. See also Supplementary Table 1.

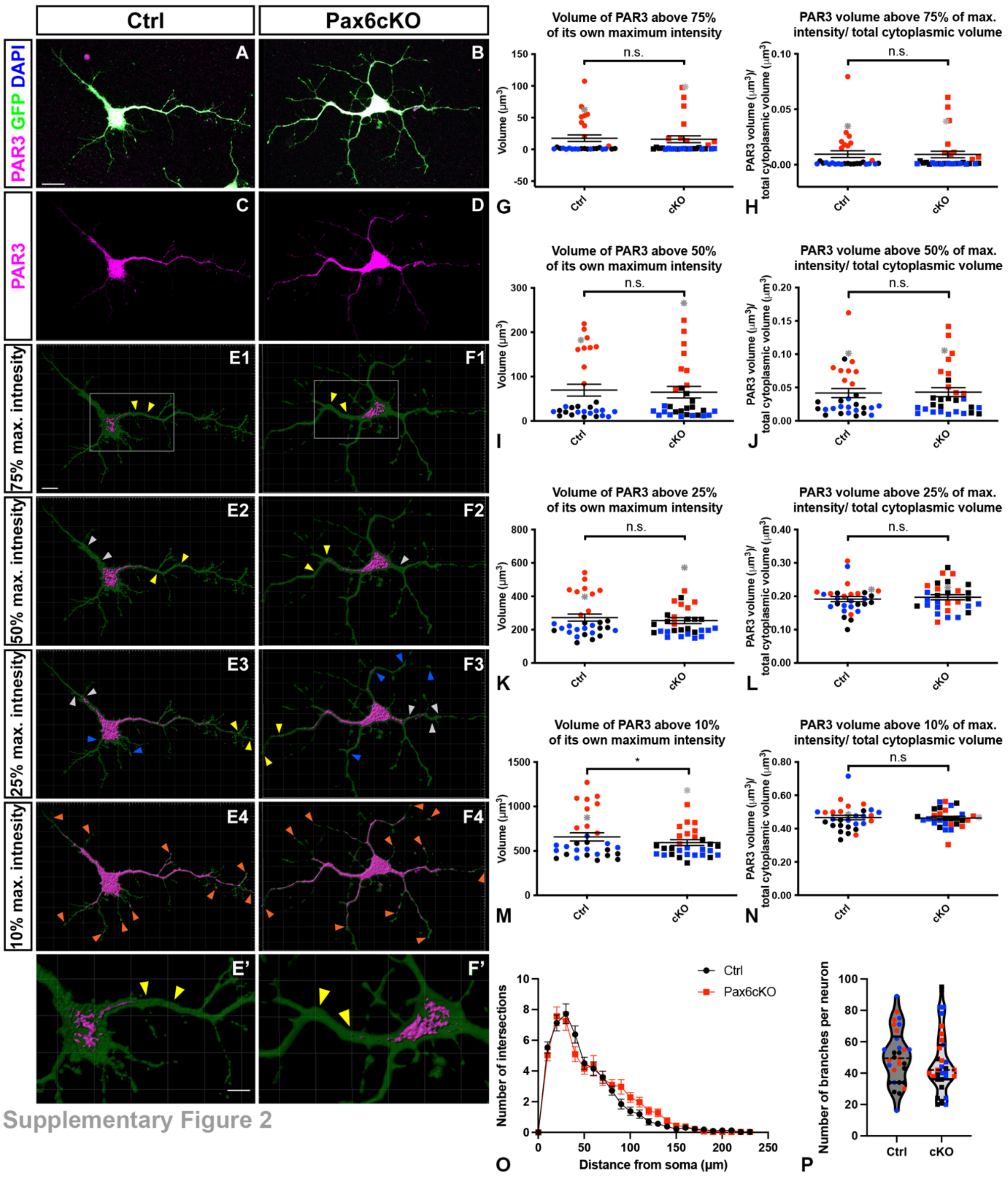

Fig. S2. Distribution of Par3 proteins and neurite branching of the developing prethalamic neurons at 3DIV. (A, B) Prethalamic neurons from the Ctrl (A) and Pax6cKO (B) embryos were cultured for 3DIV and stained for Par3, GFP and DAPI. **(C, D)** Par3 expression within the cytoplasm of the neurons. **(E1-F1)** Par3 expression within the cytoplasm when the intensity threshold was set at 75% of the maximum intensity of the Par3 channel. The majority of the Par3 volumes were distributed within the soma. However, Par3 volumes were also found in the stem of the longest neurites (yellow arrowheads). **(E2-F2)** Par3 expression within the cytoplasm when the intensity threshold was set at 50% of the maximum intensity of the Par3 channel.

More Par3 volumes were detected in the soma, the stem of the longest neurites (yellow arrowheads), as well as in other neurites (grey arrowheads). **(E3-F3)** Par3 expression within the cytoplasm when the intensity threshold was set at 25% of the maximum intensity of the Par3 channel. Par3 volumes within the cytoplasm continued to increase. Par3 volumes could be found at the tip of the longest neurites (yellow arrowheads) and other neurites (grey and blue arrowheads). **(E4-F4)** Par3 expression within the cytoplasm when the intensity threshold was set at 10% of the maximum intensity of the Par3 channel. PAR3 volumes had filled up the cytoplasm and could be found at almost all the tip of the neurites and protrusions (orange arrowheads). **(E'-F')** Zoom in on the boxed area in E1 and F1. Scale bars: A-D, 15µm, E1-F5, 10µm, E'-F', 5µm. **(G-N)** Comparison of Par3 volumes and percentage of Par3 volumes against the cytoplasmic volume between the Ctrl and the Pax6cKO prethalamic neurons. Each dot (data point) represented the value of Par3 volume (G, I, K, M) or Par3 volume/ total cytoplasmic volume (H, J, L, N) from each neuron at a specific threshold. Data derived from the corresponding images in panels A-F are shown as grey asterisks in panels G-N. 10 neurons with the Ctrl and Pax6cKO genotypes from each of 3 cultures from separate litters were measured. Mixed-effect model. Mean ± SEM. *: $p<0.05$. The data points of the neurons from the same litter were marked with the same colour. **(O)** Sholl analysis showed no significant difference in the number of neurite intersections between 10 and 240 µm from the soma between the Pax6cKO and the control prethalamic neurons, $p=0.194$. **(P)** Evaluation of number of branches per neuron showed no significant difference between the Pax6cKO and the control prethalamic neurons, $p=0.238$. 10 neurons with the Ctrl and Pax6cKO genotypes from each of 3 cultures from separate litters were measured. Mixed-effect model. Mean ± SEM.

Development • Supplementary information

Table S1. List of significantly up- and downregulated (adjusted p<0.05) genes and GO terms in the E13.5 prethalamus after induced acute Pax6 deletion at E9.5. Related to Figure 1.

Genes	LFC	P value	Description
**GEFs for Rac**			
Dock4	1.03	3.39E-10	dedicator of cytokinesis 4
Dock2	0.86	0.00196	dedicator of cytokinesis 2
Dock3	0.68	0.00300	dedicator of cytokinesis 3
Dock9	0.66	0.00671	dedicator of cytokinesis 9
Dock10	0.76	0.00339	dedicator of cytokinesis 10
Dock8	0.95	0.00178	dedicator of cytokinesis 8
**Scaffold for GEFs**			
Elmo1	0.87	2.21E-10	engulfment and cell motility 1
**Scaffold for CDC42**			
Fgd1	0.57	0.00362	FYVE, RhoGEF and PH domain containing 1
Fgd5	1.04	3.11E-05	FYVE, RhoGEF and PH domain containing 5
Fgd2	0.67	0.00234	FYVE, RhoGEF and PH domain containing 2
Fgd6	0.65	0.00503	FYVE, RhoGEF and PH domain containing 6
**GAP for Rho and Rac**			
Srgap3	0.92	0.00086	SLIT-ROBO Rho GTPase activating protein 3
**Rac**			
Rnd1	0.77	2.55E-07	Rho family GTPase 1
**Actin-bundling**			
Ablim1	0.59	0.00253	actin-binding LIM protein 1
Ablim3	1.19	7.20E-07	actin binding LIM protein family, member 3
Ablim2	0.91	0.00342	actin-binding LIM protein 2
Strip2	1.80	4.32E-07	striatin interacting protein 2
**Anti-capping for actin**			
Enah	0.67	0.00391	enabled homolog (Drosophila)
**Membrane curvature**			
Pacsin1	0.81	0.00546	protein kinase C and casein kinase substrate in neurons 1
**Microtubule polymerisation/bundling**			
Mapt	1.16	4.90E-08	microtubule-associated protein tau
Map6	0.70	0.00732	microtubule-associated protein 6
Map9	0.60	0.00746	microtubule-associated protein 9
Map2	0.32	0.01108	microtubule-associated protein 2
Mark1	0.78	0.00014	MAP/microtubule affinity-regulating kinase 1
Mark4	0.76	0.00720	MAP/microtubule affinity-regulating kinase 4
Apc2	0.86	0.00485	adenomatosis polyposis coli 2
Apc	0.78	0.00820	adenomatosis polyposis coli

Crmp1	0.32	0.00312	collapsin response mediator protein 1
Dpysl5	0.66	0.00500	dihydropyrimidinase-like 5
**Par3/6 complex component**			
Prkcz	0.37	0.00431	protein kinase C, zeta
Kif3a	0.45	0.00484	kinesin family member 3A
Tiam1	0.66	0.00821	T cell lymphoma invasion and metastasis 1
Arhgap35	0.59	0.01060	Rho GTPase activating protein 35
Smurf1	0.53	0.00328	SMAD specific E3 ubiquitin protein ligase 1
Pard3b	0.80	0.00822	par-3 family cell polarity regulator beta
Apc	0.78	0.00820	adenomatosis polyposis coli
**Cytoskeleton protein in the axon initial segment**			
Ank3	1.19	0.00105	ankyrin 3, epithelial
Sptb	1.17	0.00054	spectrin beta, erythrocytic
Sptbn1	1.03	0.00096	spectrin beta, non-erythrocytic 1
Sptbn4	1.36	4.52E-06	spectrin beta, non-erythrocytic 4
**Voltage-gated Sodium channels (VGSCs)**			
Scn1a	0.77	0.00123	sodium channel, voltage-gated, type I, alpha
Scn3b	0.72	2.96E-06	sodium channel, voltage-gated, type III, beta
Scn2b	0.58	0.00118	sodium channel, voltage-gated, type II, beta
Scn3a	0.39	0.00502	sodium channel, voltage-gated, type III, alpha
**Rapidly inactivating A-type K+ channel**			
Kcna4	1.87	1.40E-16	potassium voltage-gated channel, shaker-related subfamily, member 4
Kcnc3	1.02	0.00040	potassium voltage gated channel, Shaw-related subfamily, member 3
Kcnc4	0.79	0.00252	potassium voltage gated channel, Shaw-related subfamily, member 4
Kcnd1	0.61	0.00769	potassium voltage-gated channel, Shal-related family, member 1
Kcnd3	1.02	3.78E-10	potassium voltage-gated channel, Shal-related family, member 3
Kcnd2	0.84	9.81E-07	potassium voltage-gated channel, Shal-related family, member 2
**Voltage-gated Ca2+ channels**			
Cacna1e	1.09	0.00012	calcium channel, voltage-dependent, R type, alpha 1E subunit
Cacna2d2	0.68	0.00930	calcium channel, voltage-dependent, alpha 2/delta subunit 2
Cacna1a	0.61	0.00114	calcium channel, voltage-dependent, P/Q type, alpha 1A subunit
Cacna2d4	1.87	2.98E-08	calcium channel, voltage-dependent, alpha 2/delta subunit 4
Cacna1g	1.21	4.11E-07	calcium channel, voltage-dependent, T type, alpha 1G subunit
**Hyperpolarization-activated cyclic nucleotide–gated (HCN) channels**			
Hcn2	0.95	1.12E-05	hyperpolarization-activated, cyclic nucleotide-gated K+ 2
Hcn4	0.70	0.00481	hyperpolarization-activated, cyclic nucleotide-gated K+ 4
Hcn3	0.40	0.00852	hyperpolarization-activated, cyclic nucleotide-gated K+ 3

Table S2. List of genes found in the neuronal morphogenesis and ion transport GO terms that encode proteins with specific neuronal functions. LFC, log-fold change.

Click here to download Table S2

Development • Supplementary information

Table S3. Key resources

Reagent or resource	Source	Identifier
**Antibodies**		
Rabbit polyclonal anti-Pax6 (1:200)	BioLegend	Cat#901301, RRID: AB_2565003
Mouse monoclonal anti-Pax6 (1:10)	A gift from Prof. V van Heyningen, AD2.38 Simpson et al., 2009	N/A
Mouse monoclonal anti-Beta III tubulin (Tuj1) (1:200)	Abcam	Cat# ab18207, RRID: AB_444319
Mouse monoclonal anti-Ankyrin-G, clone N106/36 (1:200)	Antibodies Inc.	Cat# 75-146
Monoclonal Anti-Sodium Channel, Pan antibody produced in mouse (1:100)	Sigma-Aldrich	Cat# S8809-.1MG
Rabbit polyclonal Anti-Partitioning-defective 3 Antibody (1:100)	Millipore	Cat# 07-330
Goat polyclonal Anti-GFP antibody (1:200)	Abcam	Cat# ab6673
Chicken polyclonal Anti-GFP antibody (1:200)	Abcam	Cat# ab13970
Goat anti-mouse biotinylated secondary antibody (1:200)	Vector laboratories	Cat# BA-9200, RRID:AB_2336171
Goat anti-rabbit biotinylated secondary antibody (1:200)	Vector laboratories	Cat# BA-1000, RRID:AB_2313606
Donkey anti-mouse Alexa 568 secondary antibody (1:200)	Thermo Fisher Scientific	Cat# A10037, RRID:AB_2534013
Donkey anti-rabbit Alexa 647 secondary antibody (1:200)	Invitrogen	Cat # A-31573
Donkey anti-chicken Alexa 488 secondary antibody (1:200)	Stratech Scientific Limited	Cat# 703-545-155-JIR
Donkey anti-rabbit Alexa 568 secondary antibody (1:200)	Invitrogen	Cat # A10042
Donkey anti-goat Alexa 488 secondary antibody (1:200)	Invitrogen	Cat # A-11055
Donkey anti-rat Alexa 488 secondary antibody (1:200)	Thermo Fisher Scientific	Cat# A-21208, RRID:AB_141709
Streptavidin, Alexa Fluor 488 conjugate antibody (1:200)	Thermo Fisher Scientific	Cat# S11223, RRID:AB_2336881
**Chemicals, Peptides, and Recombinant Proteins**		
Tamoxifen	Sigma-Aldrich	|||T5648; CAS: 10540-29-1
**Critical Commercial Assays**		

Reagent or resource	Source	Identifier
DAB peroxidase substrate kit	Vector Laboratories	SK4100
Papain Dissociation System	Worthington Biochemical Corp	Cat# LK003150
**Experimental Models: Organisms/Strains**		
Mouse: CAGGCre-ERTM; RCE (GFP)	This paper Hayashi and McMahon, 2002; Sousa et al., 2009	N/A
Mouse: Pax6loxP	Simpson et al., 2009	N/A
**Software and Algorithms**		
Graphpad Prism 8	Graphpad software	
pClamp	Molecular Devices	
Matlab_R2018b	Mathworks	
Fiji (Image J)	Schindelin et al., 2012	https://fiji.sc/
IMARIS 9.1.2	Bitplane	

Development • Supplementary information
