# Supplementary material for: Pax6 loss alters the morphological and electrophysiological development of mouse prethalamic neurons
Source: Development. 2022 Mar 17;149(6):dev200052. doi: 10.1242/dev.200052 (PMC8977098; doi:10.1242/dev.200052)
Supplement: Reviewer comments [file develop-149-200052_review_history.pdf]

Pax6 loss alters the morphological and electrophysiological development of mouse prethalamic neurons

Tian Tian, Idoia Quintana-Urzainqui, Zrinko Kozic, Thomas Pratt and David J. Price
DOI: 10.1242/dev.200052

Editor: Paola Arlotta

Review timeline

Original submission:	26 July 2021
Editorial decision:	17 September 2021
First revision received:	25 January 2022
Accepted:	16 February 2022

Original submission

First decision letter

MS ID#: DEVELOP/2021/200052

MS TITLE: Pax6 loss alters the morphological and electrophysiological development of mouse prethalamic neurons

AUTHORS: Tian Tian, Idoia Quintana-Urzainqui, Zrinko Kozic, Thomas Pratt, and David J. Price

I am sorry that it took so long to get the reviewers' comments. I have now received all the referees' reports on the above manuscript, and have reached a decision. The referees' comments are appended below, or you can access them online: please go to BenchPress and click on the 'Manuscripts with Decisions' queue in the Author Area.

As you will see, the referees express considerable interest in your work, but have some significant criticisms and recommend a substantial revision of your manuscript before we can consider publication. If you are able to revise the manuscript along the lines suggested, which may involve further experiments, I will be happy receive a revised version of the manuscript. Your revised paper will be re-reviewed by one or more of the original referees, and acceptance of your manuscript will depend on your addressing satisfactorily the reviewers' major concerns. Please also note that Development will normally permit only one round of major revision.

We are aware that you may be experiencing disruption to the normal running of your lab that make experimental revisions challenging. If it would be helpful, we encourage you to contact us to discuss your revision in greater detail. Please send us a point-by-point response indicating where you are able to address concerns raised (either experimentally or by changes to the text) and where you will not be able to do so within the normal timeframe of a revision. We will then provide further guidance. Please also note that we are happy to extend revision timeframes as necessary.

Please attend to all of the reviewers' comments and ensure that you clearly highlight all changes made in the revised manuscript. Please avoid using 'Tracked changes' in Word files as these are lost in PDF conversion. I should be grateful if you would also provide a point-by-point response detailing how you have dealt with the points raised by the reviewers in the 'Response to Reviewers' box. If you do not agree with any of their criticisms or suggestions please explain clearly why this is so.

© 2022. Published by The Company of Biologists under the terms of the Creative Commons Attribution License (https://creativecommons.org/licenses/by/4.0/).

Reviewer 1

Advance summary and potential significance to field

Pax6 is a key regulator of brain development and serves multifunctional roles in many aspects. In this study, the authors further extend their research on the function of Pax6 in prethalamic regions by using conditional knockout of the gene in mice. They performed various morphological and electrophysiological assays using cultured neurons derived from the prethalamic nucleus. They found that conditional (stage-specific) deletion of Pax6 affected neurite elongation, positions of axon initial segments, and physiological properties. Their findings are very important considering the evidence that human PAX6 gene is responsible for syndromic autism spectrum disorder (see SFARI database), which is not unfortunately considered (e.g., Kikkawa et al., Brain Res, 2019 and references therein). Actually, the thalamus is a pivotal brain region to coordinate various sensory input, and many autistic patients suffers sensory perception. Their strategy to delete Pax6 function is not brain-region specific, but developmental stage-dependent by injecting tamoxifen into Pax6-floxed mice. Therefore, there could be some indirect effect come from the loss-of-functions of the cortical neurons when they lost Pax6 function at the time of neural progenitor stage; Pax6 turns off in cortical neurons. However, since there is less study on the thalamus development among other brain regions, this reviewer would imagine that their work will be remarkable in the field. To be accepted in Development, this reviewer would think that the analyses are not comprehensive in regard with the synaptic functions; they should perform more assays as suggested below.

Comments for the author

1) It is critical to know how loss of Pax6 affect neuronal functions in the prethalamus in vivo. Therefore, morphology of neuronal connections and expression of synaptic molecules should be examined. Ideally, it would be the best to observe pre- and post-synaptic molecules of prethalamic axons/dendrites at the super-resolution level using STED microscope and see the difference between Pax6 cKO and the wild type mice. At least, such morphological analyses should be performed in cultured neurons, which might serve nice evidence to support the eletrophysiolocical data shown in Figure 4.
2) From the morphological data shown in Supplementary Figure 1, it seems that neurons derived from Pax6 cKO have more branches. This could be statistically analyzed if the pictures are representative ones. There is a standard assay to identify such difference (e.g., Thongkorn et al., Sci Rep, 2021). Furthermore, it would be faithful to indicate origins of the morphological images within the statistic plots by using different color for the specific dots.
3) It would be better to see the formation of synapses even in vitro. Dissociated culture is a very artificial condition.
4) To understand the segmental composition within prethalamic axons, the authors should observe key molecules for axon initial segments (AISs) other than AnkyrinG, spectrin voltage gated sodium channels (see e.g., Haung and Rasband, Ann N Y Acad Sci, 2018).
Then, there would be more discussion in regard with neurological/psychiatric diseases.
5) Scale bars are needed for Figure 1B, 1B’, and 3A-3D.

Reviewer 2

Advance summary and potential significance to field

Tian and colleagues aimed to study the extent to which the actions of Pax6 influence the maturation of postmitotic neurons in the prethalamus of mice. Pax6 is expressed throughout the neuroepithelium of the forebrain and animal models with constitutive deletions of Pax6 preclude the analysis its function in post-mitotic cells because it plays an important role also earlier in development, in the specification of the neuroepithelium. To avoid this issue, the authors conducted their experiments in an model (already published) wherein Pax6 is conditionally deleted from prethalamic cells from E11.5 onwards using a tamoxifen-based strategy. They cultured E13.5 prethalamic cells up to 9 DIV and found abnormalities in the Pax6-deleted cells in some features that could be predicted from the altered genetic profile found in a previous RNAseq. They found abnormalities in dynamics of the extension of neurites, in the structure of the axon initial segment, and in the waveform of the action potential and the excitability of the cells.

© 2022. Published by The Company of Biologists under the terms of the Creative Commons Attribution License (https://creativecommons.org/licenses/by/4.0/).

Comments for the author

The research question is well-presented and relevant. However, the result fell short on expectations derived from the authors' purpose. The study needs more experimental data to create a basis to convince the reader that the hypothesis has been tested and conclusions were properly raised. The authors did not overstate the conclusions, they claim what they found, but I think that the they need an extended corpus of results to turn this manuscript appropriate for publication in Development. Most of the differences found are subtle, and difficult to link one with other. So, it seems a good starting point but I think the authors need to add experimental evidences to build a stronger story.
Some suggestions are: to use slices in parallel to the cultures to show some anatomical and functional consequences; ii) to track the development of these cells (and the derived structures), checking morphology, physiology, connectivity throughout development; iii) to look for a link between Pax6 and the abnormalities found.
Apart from this, the manuscript has no major issues. The Introduction provides a concise and clear background to the study with a satisfactory citation of prior literature. The results are well explained, and the discussion is centered on the important aspects of the results.

Other comments:
1) The data shown in Figures 1A and 1B is implicit in a previous publication of the group (Figure 1A-D in Quintana-Urzainqui et al 2018).
2) Page 5, last paragraph: "Pax6cKO prethalamic neurons had fewer neurites than Ctrl neurons at 1DIV but not after longer culture (Figure 2M)"
Comment: The authors do not provide in the text the magnitudes of the quantifications of Fig 2M, nor do they show us the number of experimental units (N) used. Looking at the graph in Fig 2M, the actual average number of neurites at 1DIV seems to be 2.7 and 2.4 in control and Pax6cKO respectively. The authors report that this difference is significant, however, its biological relevance seems minimal. Mostly, knowing that in the following days the statistical difference disappears. In any case, the authors should add the number of experimental units (we need to know who is the experimental unit: cells, wells, cultures, ...), statistical tests and corrections used in the analysis, as well as P-values. Here and elsewhere in the manuscript.
Moreover, the Y-axis should start at 0 in Fig 2M.
3) Page 9, last paragraph: "Mutant prethalamic neurons show abnormal axonal extension"
Comment: As the differences are subtle, this statement seems misleading.
4) Which proportion of prethalamic cells are Pax6+?

<u>Reviewer 3</u>

Advance summary and potential significance to field

In the manuscript entitled 'Pax6 loss alters the morphological and electrophysiological development of mouse prethalamic neurons' Tian and collaborators studied the role of Pax6 in the morphological and electrophysiological development of prethalamic neurons in vitro.

The study is based on the re-examination of previously published RNA-seq data from the E13.5 mouse prethalamic area after acute Pax6 knockout induced on E9.5 (Quintana-Urzainqui et al., 2018). Among up- or downregulated genes authors found several who's GO terms related to neuronal morphogenesis and ion transport. Authors hypothesised that conditional Pax6 knockout affects the development of prethalamic neuronal morphology, the formation of the AIS (axon initial segments) and the activity of prethalamic neurons. Tian and collaborators tested these hypotheses within primary in vitro cultures of Pax6fl/+ (Ctrl) and Pax6fl/fl (cKO) prethalamic neurons. Depending on the exact day of cell culture, Authors found small but statistically significant differences in the length of prethalamic neurites, which suggests deregulation in the mechanisms that affect final axon length. Later Tian and collaborators shown that cKO prethalamic neurons abnormally form AIS, which are longer and further from the Ctrl cell somas. Last, authors performed whole-cell patch-clamp on the cultured prethalamic neurons, revealing anormal excitability and waveform of the action potentials in Pax6 cKO neurons.

© 2022. Published by The Company of Biologists under the terms of the Creative Commons Attribution License (https://creativecommons.org/licenses/by/4.0/).

The manuscript is written clearly and concisely and the experiments are well-executed. Study does not provide an in-depth exploration of the molecular mechanisms directly responsible for the observed phenotypes. That said, the study provides interesting new evidence regarding the role of Pax6 in postmitotic prethalamic neurons and as a whole sets ground for further detailed molecular and in vivo analyses. As the intrinsic mechanisms of morphological and functional maturation of neurons in many subcortical structures are still poorly characterised, a study like this provides valuable cues for future research. In summary, the conclusions made by the authors are well drawn by their experimental data and the manuscript deserves publication after a minor revision.

Comments for the author

Suggestions to authors:
1. Description in the Materials and Methods suggests that the Authors isolated prethalamus from E13.5 embryos using solely morphological landmarks. That brings up a question about the precision of the procedure as at this age the prethalamic region is very small and can be easily contaminated with cells from surrounding structures. That said, the appearance of a GFP marker on Figure 3 and in the ‘Measurement of PAR3 distribution’ in Materials and Methods suggests that in the isolation Authors were additionally guided by the GFP expression, as described in (Quintana-Urzainqui et al., 2018). If so, I believe this should be better stated in the manuscript, as it explains how the Authors were able to discriminate between prethalamic and contaminant cells in their in vitro cell cultures.
2. I would appreciate if Authors restructured the figures so that the panels are addressed in the same order as they are presented, as sometimes this is not the case. Examples: Figure 1C-D (page 4) is mentioned prior to figure 1B-B’ (page 5); Figure 2A-E (page 10) is mentioned long after Figure 2F-L’ (page 5); Figure 2G-G’ is mentioned before Figure 2F-F’.
3. Figure 3A-D present the fluorescence of AnkG/VGSC, GFP, PAX6 and DAPI. Assuming that PAX6 staining is presented in white, there is little to no difference in PAX6 level in Ctrl and cKO cells. Possibly the lack of a clear PAX6 staining arises from the presentation of too many fluorophores which masks the proper PAX6 signal. Authors should present stainings that clearly show presence of PAX6 in their Ctrl neuronal cultures and loss of Pax6 in cKO neuronal cultures.

First revision

<u>Author response to reviewers' comments</u>

We thank our reviewers for their time and valuable feedback. We were very encouraged to see that they found that our study ‘will be remarkable in the field’ (R1), that ‘the research question is well-presented and relevant’ and ‘a good startingpoint’ (R2) and ‘the manuscript is written clearly and concisely and the experiments are well-executed’ and ‘a study like this provides valuable cues for future research’ (R3). We appreciate the suggestions for further experiments from Reviewers 1 and 2. As we explain below, we have carried out the additional work that was feasible inthe context of a revision of our existing study. We would love to extend the work in the new directions suggested in the future.

REVIEWER 1
“To be accepted in Development, this reviewer would think that the analyses are not comprehensive in regard with the synaptic functions.”

1) It is critical to know how loss of Pax6 affect neuronal functions in the prethalamusin vivo. Therefore, morphology of neuronal connections and expression of synaptic molecules should be examined. Ideally, it would be the best to observe pre- and post-synaptic molecules of prethalamic axons/dendrites at the super-resolution levelusing STED microscope and see the difference between Pax6cKO and the wild typemice. At least, such morphological analyses should be performed in cultured neurons, which might serve nice evidence to support the electrophysiological data shown in Figure 4.

© 2022. Published by The Company of Biologists under the terms of the Creative Commons Attribution License (https://creativecommons.org/licenses/by/4.0/).

We agree with Reviewer 1 that synaptic functions would be an interesting aspect to investigate. However, this would require an extensive new study that is not directly related to the questions we posed and the findings reported in our current manuscript. There is no possibility of completing such a study in a timescale normally associated with manuscript revision. It would be very doubtful that the shortformat of the existing manuscript would be adequate for such a study. Reviewer 1 justified their suggestion to perform the synaptic morphological analyses in cultured neurons on the basis that it might provide nice evidence in support of the electrophysiological data. It would not, however, provide direct support for our *existing* data because they concern the *intrinsic* properties of the neurons rather than synaptic function. In summary, the work proposed by the Reviewer would extend ourwork in interesting ways rather than support our existing findings.

2) From the morphological data shown in Supplementary Figure 1, it seems that neurons derived from Pax6 cKO have more branches. This could be statistically analyzed if the pictures are representative ones. There is a standard assay to identify such difference (e.g., Thongkorn et al., Sci Rep, 2021). Furthermore, it wouldbe faithful to indicate origins of the morphological images within the statistic plots by using different colour for the specific dots.

We thank the reviewer for this suggestion and have now conducted the assay as proposed and have included the new data in Supplementary Figure 2O, P. We did not find any effect of genotype on branching. We have also revised the graphs in Supplementary Figure 2G-N to indicate data derived from the corresponding imagesin panels A-E (shown as grey asterisks).

3) It would be better to see the formation of synapses even in vitro. Dissociatedculture is a very artificial condition.

This is essentially the same suggestion as in point (1) above. Please refer to ourresponse above.

4) To understand the segmental composition within prethalamic axons, the authors should observe key molecules for axon initial segments (AISs) other than AnkyrinG,spectrin, voltage gated sodium channels (see e.g., Haung and Rasband, Ann N Y Acad Sci, 2018). Then, there would be more discussion in regard with neurological/psychiatric diseases.

We chose to quantify AnkyrinG and voltage gated sodium channels as they are the most commonly used markers of the axon initial segment (some examples are cited in our manuscript: Grubb and Burrone, 2010; Kaphzan et al., 2011; Booker et al., 2020) and they are quite highly upregulated in our RNAseq data. We chose AnkG to represent the cytoskeletal aspect of the AIS and we chose voltage gated sodium channels to represent more of the functional aspect of the AIS. Many other published studies used only AnkG, and/or voltage gated sodium channels to mark the AIS and to show the changes of AIS under different conditions or in psychiatric diseases. TheAIS is a complex structure comprising various molecules with different functions and looking further into that complexity might be interesting but would be additional and would not affect our current conclusion. We are happy to see that Reviewer 1 recognises the potential implication of our findings for neurological/psychiatric diseases or disorders such as syndromic autism spectrum disorder and we are mosthappy to cite Kikkawa et al., Brain Res, 2019 in the revised manuscript as suggestedby Reviewer 1. However, our study does not aim to provide major insight into these conditions.

5) Scale bars are needed for Figure 1B, 1B’, and 3A-3D.

We have added the scale bars to these figures.

REVIEWER 2

Some suggestions are: to use slices in parallel to the cultures to show some anatomical and functional consequences; ii) to track the development of these cells(and the derived structures), checking morphology, physiology, connectivity throughout development; iii) to look for a link between Pax6 and the abnormalities found.

We think the experiments proposed here would need an entirely new study well beyond the scope of our current manuscript and are, therefore, not feasible in the context of a revision. We chose

© 2022. Published by The Company of Biologists under the terms of the Creative Commons Attribution License (https://creativecommons.org/licenses/by/4.0/).

the in vitro dissociated culture of prethalamic neurons to better identify the cell autonomous effect of Pax6 deletion on the development of prethalamic cells in the post-mitotic stage, given the various defectsin cortical and diencephalic development Pax6 deletions cause, as mentioned and cited in our manuscript. Just as Reviewer 2 mentioned, our study 'seems a good starting point' and we think that future studies utilising an in vivo or ex vivo setting would bring more complementary insight and help complete the bigger picture.

2) Page 5, last paragraph: "Pax6cKO prethalamic neurons had fewer neurites than Ctrl neurons at 1DIV but not after longer culture (Figure 2M)" Comment: The authorsdo not provide in the text the magnitudes of the quantifications of Fig 2M, nor do theyshow us the number of experimental units (N) used. Looking at the graph in Fig 2M, the actual average number of neurites at 1DIV seems to be 2.7 and 2.4 in control and Pax6cKO respectively. The authors report that this difference is significant, however, its biological relevance seems minimal. Mostly, knowing that in the following days the statistical difference disappears. In any case, the authors should add the number of experimental units (we need to know who is the experimental unit:cells, wells, cultures, ...), statistical tests and corrections used in the analysis, as wellas P-values. Here and elsewhere in the manuscript. Moreover, the Y-axis should start at 0 in Fig 2M.

We have edited the manuscript to make clear the statistical tests used (mixed-effectsmodels), p and n values (now stated in the text, as requested) and the definition of n (also now stated in the text at first usage) We have revised the Y-axis in Figure 2M as requested.

4) Which proportion of prethalamic cells are Pax6+?

We think that most, if not all, progenitor cells in the prethalamus are Pax6-positive. After neurogenesis, a large population of the prethalamic post-mitotic cells retain theexpression of Pax6 (Duan, Deyi, et al. Brain Structure and Function 218.2 (2013): 353-372.) We have made this clear in the revision.

REVIEWER 3

1. Description in the Materials and Methods suggests that the Authors isolated prethalamus from E13.5 embryos using solely morphological landmarks. That bringsup a question about the precision of the procedure, as at this age the prethalamic region is very small and can be easily contaminated with cells from surrounding structures. That said, the appearance of a GFP marker on Figure 3 and in the 'Measurement of PAR3 distribution' in Materials and Methods suggests that in the isolation Authors were additionally guided by the GFP expression, as described in (Quintana-Urzainqui et al., 2018). If so, I believe this should be better stated in themanuscript, as it explains how the Authors were able to discriminate between prethalamic and contaminant cells in their in vitro cell cultures.

We have added to the description of this in Materials and Methods to make clearerwhat we did to make out dissections as accurate as possible.

2. I would appreciate if Authors restructured the figures so that the panels are addressed in the same order as they are presented, as sometimes this is not the case. Examples: Figure 1C-D (page 4) is mentioned prior to figure 1B-B' (page 5); Figure 2A-E (page 10) is mentioned long after Figure 2F-L' (page 5); Figure 2G-G' ismentioned before Figure 2F-F'.

We have addressed this in the revision.

3. Figure 3A-D present the fluorescence of AnkG/VGSC, GFP, PAX6 and DAPI. Assuming that PAX6 staining is presented in white, there is little to no difference in PAX6 level in Ctrl and cKO cells. Possibly the lack of a clear PAX6 staining arises from the presentation of too many fluorophores, which masks the proper PAX6 signal. Authors should present stainings that clearly show presence of PAX6 in theirCtrl neuronal cultures and loss of Pax6 in cKO neuronal cultures.

We have clarified this in the revised manuscript. In these experiments we found that all neurons of both genotypes reacted with an antibody that recognizes an epitope inthe C-terminal domain of Pax6 (Biolegend, Cat #901301; referred to in Fig. 3A-D as Pax6-C), which is produced even in

© 2022. Published by The Company of Biologists under the terms of the Creative Commons Attribution License (https://creativecommons.org/licenses/by/4.0/).

Pax6cKO neurons due to translation from preserved internal initiation sites (see Methods; Simpson, T. Ian, et al. Neural development 4.1 (2009): 1-18; Kammandel, Birgitta, et al. Developmental biology 205.1 (1999): 79-97.). This confirmed that we were comparing Ctrl and Pax6cKOneurons that expressed the *Pax6* gene (although the latter did not generate full- length functional Pax6 protein: Supplementary Figure 1B, B').

Second decision letter

MS ID#: DEVELOP/2021/200052

MS TITLE: Pax6 loss alters the morphological and electrophysiological development of mouse prethalamic neurons

AUTHORS: Tian Tian, Idoia Quintana-Urzainqui, Zrinko Kozic, Thomas Pratt, and David J. Price
ARTICLE TYPE: Research Report

I am happy to tell you that your manuscript has been accepted for publication in Development, pending our standard ethics checks.

Reviewer 2

Advance summary and potential significance to field

Tian and collaborators assess the function of Pax6 in developing prethalamic neurons. Using conditional knockout mice, the authors delete PAX6 at the specific developmental stage at which it is expressed in prethalamic neurons. Guided by the genetic profile observed in previous RNAseq data, the authors focus their analysis on the morphological and physiological properties of control and Pax-negative immature neurons of the prethalamus. Using cultures of dissociated neurons to assess cell-autonomous effects, they found subtle but statistically significant differences in neurite length, the structure of the axon initial segment, the cell excitability and the waveform of the action potential. These features are key for the normal function of neurons and networks. Therefore, I reflect that the aim of this manuscript is relevant and of interest for developmental neurobiologists.

Comments for the author

In the revised version, the authors did not add new experimental data but they performed modifications that improve the quality of the manuscript. It is a bit disappointing that the authors did not try to include new experimental evidence. I suggested some experiments (Reviewer 1 too) that the authors considered out of their scope. I accept their reasons. At the same time, I want to clarify that my aim was to provide some ideas that may inspire them to strengthen the manuscript and reach with no doubts the standards of Development.
However, the authors addressed the other concerns of the reviewers and improved the appearance and quality of the manuscript. The authors reorganized the figures to highlight the original findings and align them with the main text. They provide now details about the statistical analysis that add rigor to the evidence (nevertheless, as internal controls for the authors, I recommend a power analysis of the data). Also, the authors rephrased some of their statements to match evidence and conclusions. In general, I still think that this work seems a good starting point, but the current version is solid, with potential for future in-depth explorations, and could be considered for publication.

© 2022. Published by The Company of Biologists under the terms of the Creative Commons Attribution License (https://creativecommons.org/licenses/by/4.0/).

Reviewer 3

Advance summary and potential significance to field

In the manuscript entitled 'Pax6 loss alters the morphological and electrophysiological development of mouse prethalamic neurons' Tian and collaborators studied the role of Pax6 in the morphological and electrophysiological development of prethalamic neurons in vitro.

The study is based on the re-examination of previously published RNA-seq data from the E13.5 mouse prethalamic area after acute Pax6 knockout induced on E9.5 (Quintana-Urzainqui et al., 2018). Among up- or downregulated genes authors found several who's GO terms related to neuronal morphogenesis and ion transport. Authors hypothesised that conditional Pax6 knockout affects the development of prethalamic neuronal morphology, the formation of the AIS (axon initial segments) and the activity of prethalamic neurons. Tian and collaborators tested these hypotheses within primary in vitro cultures of Pax6fl/+ (Ctrl) and Pax6fl/fl (cKO) prethalamic neurons. Depending on the exact day of cell culture, Authors found small but statistically significant differences in the length of prethalamic neurites, which suggests deregulation in the mechanisms that affect final axon length. Later Tian and collaborators shown that cKO prethalamic neurons abnormally form AIS, which are longer and further from the Ctrl cell somas. Last, authors performed whole-cell patch-clamp on the cultured prethalamic neurons, revealing anormal excitability and waveform of the action potentials in Pax6 cKO neurons.

The manuscript is written clearly and concisely and the experiments are well-executed. Study does not provide an in-depth exploration of the molecular mechanisms directly responsible for the observed phenotypes. That said, the study provides interesting new evidence regarding the role of Pax6 in postmitotic prethalamic neurons and as a whole sets ground for further detailed molecular and in vivo analyses. As the intrinsic mechanisms of morphological and functional maturation of neurons in many subcortical structures are still poorly characterised, a study like this provides valuable cues for future research.

Comments for the author

Authors have adequately addressed the reviewer's comments and suggestions. In my opinion the manuscript is suitable for publication.

© 2022. Published by The Company of Biologists under the terms of the Creative Commons Attribution License (https://creativecommons.org/licenses/by/4.0/).
